# Oxidative stress triggers Itch-mediated TXNIP degradation and NF-κB activation promoting chronic obstructive pulmonary disease

**DOI:** 10.1186/s12931-025-03369-5

**Published:** 2025-10-17

**Authors:** Pei-Yun Lin, Kang-Yun Lee, Shu-Chuan Ho, Hsiao-Chi Chuang, Bing-Hua Su, Ying-Jung Wu, Po-Chun Tseng, Tsung-Ting Tsai, Chiou-Feng Lin, Rahmat Dani Satria, Fu-Chia Shih, Chia-Ling Chen

**Affiliations:** 1 Graduate Institute of Medical Sciences, College of Medicine, Taipei Medical University, Taipei , 110 Taiwan; 2 School of Respiratory Therapy, College of Medicine, Taipei Medical University, Taipei, 110 Taiwan; 3 Division of Pulmonary Medicine, Department of Internal Medicine, Shuang Ho Hospital, Taipei Medical University, New Taipei City, 235 Taiwan; 4 Division of Pulmonary Medicine, Department of Internal Medicine, School of Medicine, College of Medicine, Taipei Medical University, Taipei, 110 Taiwan; 5 TMU Research Center of Thoracic Medicine, Taipei Medical University, Taipei, 110 Taiwan; 6 Graduate Institute of Clinical Medicine, College of Medicine, Taipei Medical University, Taipei, 110 Taiwan; 7 Department of Microbiology and Immunology, School of Medicine, College of Medicine, Taipei Medical University, Taipei, Taiwan; 8 Department of Clinical Pathology and Laboratory Medicine, Faculty of Medicine, Public Health and Nursing, Universitas Gadjah Mada, Yogyakarta, 55281 Indonesia; 9 Clinical Laboratory Installation, Dr. Sardjito Central General Hospital, Yogyakarta, 55281 Indonesia; 10 Pulmonary Research Center, Wan Fang Hospital, Taipei Medical University, Taipei, Taiwan

**Keywords:** ROS, TXNIP, NF-κB, Itch, COPD

## Abstract

**Background:**

Chronic inflammatory lung diseases, including chronic obstructive pulmonary disease (COPD), are characterized by pulmonary structural changes, narrowing of the small airways, and destruction of the lung parenchyma caused by prolonged inflammation. Sustained inflammation mediated by macrophages is considered to play a critical role in COPD pathogenesis, while the inductive mechanisms of persistent inflammation remain unclear.

**Methods:**

In vitro, RAW264.7 cells were treated with cigarette smoke extract (CSE), hydrogen peroxide, and 12-O-tetradecanoylphorbol-13-acetate. Loss-of-function assays were performed using MAPK inhibitors and Itch-specific knockdown. In vivo, lung tissues from mice exposed to whole-body cigarette smoke (CS) for 12 weeks, as well as clinical samples from healthy non-smokers, a healthy smoker, and COPD patients, were analyzed.

**Results:**

Our findings demonstrated that thioredoxin-interacting protein (TXNIP) participates in CS-induced NF-κB activation in macrophages, which may contribute to pulmonary inflammation. CSE markedly inhibited TXNIP expression in RAW264.7 cells through MAPK-dependent regulation, accompanied by the induction of iNOS/NO and COX-2. The decrease in TXNIP was also detected in lung tissues and macrophages obtained from smoking mice, while higher NF-κB activation and lung inflammation occurred simultaneously. Additionally, CS-induced oxidative stress triggered MAPK-dependent proteasomal degradation of TXNIP, leading to subsequent NF-κB activation. The expression of E3 ligase Itch was elevated in smoking mouse lungs and in hydrogen peroxide-stimulated cells, whereas specific silencing Itch significantly attenuated TXNIP degradation as well as NF-κB activation. Moreover, Itch expression was increased in lung tissues, whereas TXNIP was markedly reduced in lung tissues, bronchoalveolar lavage fluid cells, and peripheral blood mononuclear cells from patients with COPD.

**Conclusion:**

Accordingly, CS-induced oxidative stress promotes Itch-mediated TXNIP degradation, leading to NF-κB-driven inflammation in macrophages and potentially contributing to COPD pathogenesis.

**Graphical abstract:**

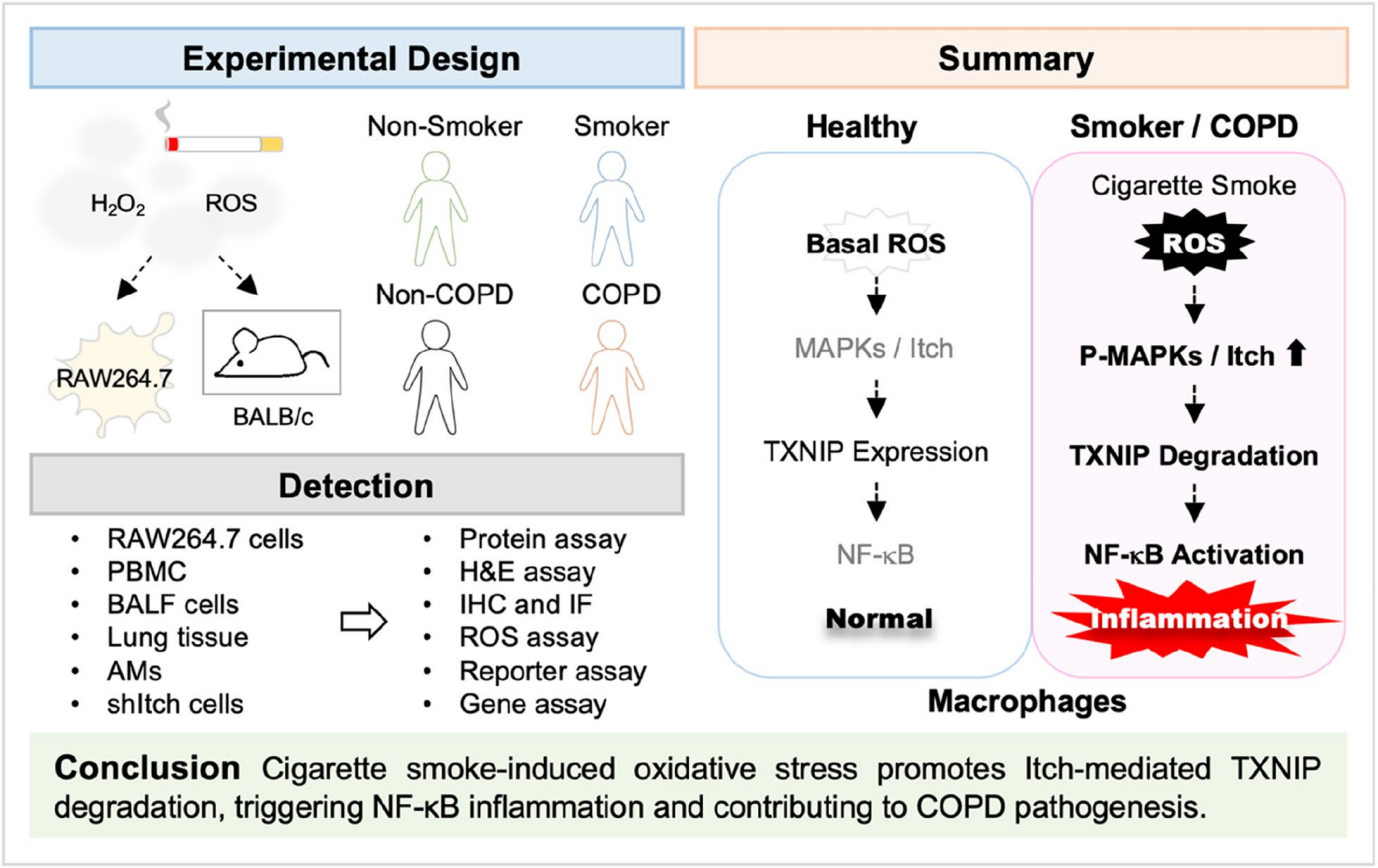

**Supplementary Information:**

The online version contains supplementary material available at 10.1186/s12931-025-03369-5.

## Introduction

Chronic obstructive pulmonary disease (COPD) is a chronic inflammatory lung disease characterized by pulmonary structural changes, narrowing of the small airways, and destruction of alveolar tissue. The most commonly encountered risk factor for COPD is tobacco usage and secondhand smoke exposure [1–3]. Cigarette smoke (CS) is a complex, dynamic and reactive mixture containing an estimated 7,000 chemicals, including reactive oxygen species (ROS) and nicotine [4]. CS-induced oxidative stress plays a vital role in causing inflammation, cell senescence, and DNA damage, which contribute to COPD pathogenesis, particularly during acute exacerbations. Increased oxidative stress activates various intracellular signaling pathways, such as the mitogen-activated protein kinases (MAPKs) and transcription factor nuclear factor (NF)-κB, which frequently interact with redox-sensitive molecular targets, thereby driving the induction of inflammatory mediators [5, 6]. So far, the effective molecular targeting of chronic and prolonged inflammation in the treatment of COPD has been widely considered. Therefore, the underlying mechanisms that control sustained inflammation need to be investigated more thoroughly.

Thioredoxin-interacting protein (TXNIP) has been originally identified as a differentially expressed gene in 1α,25-dihydroxyvitamin D3-treated HL-60 cells, also called as vitamin D3 upregulated protein 1 [7–9]. Upregulation of TXNIP is associated with diseases such as diabetes mellitus, ischemic stroke, cardiovascular diseases, and neurodegenerative disorders, while reduced TXNIP expression often correlates with the onset of tumors [8–12]. Additionally, as a member of the α-arrestin protein family, TXNIP can function as a scaffold protein (independent of its binding to TRX) in specific subcellular compartments to enhance the formation of the NLR protein 3 (NLRP3) inflammasome, promoting interleukin (IL)−1β production under oxidative stress [13], and regulate the nuclear export and degradation of hypoxia-inducible factor 1-α (HIF1-α) during hypoxia [14, 15]. Accordingly, the protein stability and gene expression of TXNIP can be regulated in response to multiple stimuli, subsequently contributing to signaling cascades that affect various biological processes.

TXNIP expression has been reported to potentially affect the development of inflammation-related lung disorders. The induction of TXNIP-mediated NLRP3 inflammasome and pyroptosis contributes to acute lung inflammation and injury in septic mice [16, 17]. Moreover, elevated TXNIP-regulated apoptosis facilitates titanium dioxide nanoparticle-exacerbated allergic airway inflammation in an ovalbumin-induced mouse model of asthma [18]. TXNIP overexpression in memory T helper (Th) 2 cells presents enhanced memory responses, including the increases of eosinophils infiltration, Th2 cytokines, and allergic airway inflammation in mice [19]. In addition, bleomycin-induced lung fibrosis develops alongside increased TXNIP/HIF1-α expression and oxidative stress in the lung tissues of rats [20]. The pathogenesis of inflammatory lung disorders is heterogeneous, and the role of TXNIP in regulating the induction of inflammation, oxidative stress, and lung disease progression, requires more investigation.

Despite the established involvement of oxidative stress and the P2 × 7/inflammasome pathway in the development and exacerbation of CS-induced COPD [5, 21], the role of TXNIP in regulating CS-associated lung inflammation and its molecular mechanisms remain unclear. In this study, we demonstrated that exposure to CS resulted in a decrease in TXNIP levels in macrophages and murine lungs through a mechanism involving the generation of ROS and the activation of MAPKs. Oxidative stress induced the expression of the Itch E3 ligase, which regulated TXNIP for degradation and subsequently led to the activation of NF-κB. Further inhibition of Itch partially mitigated TXNIP degradation and NF-κB activation. Moreover, relatively higher *ITCH* expression was detected in alveolar macrophages and small airway epithelium of cigarette smokers compared with non-smokers, whereas *TXNIP* expression was lower. TXNIP was significantly suppressed in the lung tissues, bronchoalveolar lavage fluid (BALF) cells, and peripheral blood mononuclear cells (PBMC) of COPD patients. This suggests that CS-induced oxidative stress may trigger Itch-regulated TXNIP degradation, ultimately resulting in the activation of NF-κB-mediated inflammation that potentially contributes to the development of COPD.

## Materials and methods

### Cell cultures and reagents

Murine RAW264.7 macrophages (#ATCC TIB-71™) were cultured in Dulbecco’s modified Eagle’s medium (DMEM) (#11965084, Gibco, Invitrogen, Carlsbad, CA, USA) with 10% heat-inactivated fetal bovine serum (FBS) (#04-001-1 A, Biological Industries, Kibbutz Beit Haemek, Israel) at 37 °C in a humidified atmosphere of 95% air and 5% CO2. MG132 (#1748), Lactacystin (#2267), PD98059 (#1213), SP600125 (#1496), SB203580 (#1202) are purchased from TOCRIS Bioscience (Bristol, UK). Hydrogen peroxide (#H1099), N-Acetyl-L-cysteine (NAC, #A7250), 12-O-Tetradecanoylphorbol 13-acetate (TPA, #P8139), and 4,6-diamidino-2-phenylindole (DAPI, #D9542) were purchased from Sigma-Aldrich (St Louis, MO, USA). Antibodies against phospho-ERK1/2 (Thr202/Tyr204) (#9101S), ERK1/2 (#9102S), phospho-JNK (Thr183/Tyr185) (#9251), JNK (#9252), phospho-p38 (Thr180/Tyr182) (# 9211), p38 (#9212), phospho-NF-κB (#3033), NF-κB (#4764), Itch (#12117), iNOS (#13120), COX-2 (#12282S) and the secondary antibodies HRP-conjugated goat anti-mouse (#7076S) and goat anti-rabbit (#7074S) were purchased from Cell Signaling Technology (Boston, MA, USA). Antibodies against TXNIP (#K0205-3) were purchased from MBL International Corporation (Woburn, MA, USA). Antibodies against hydroxynonenal (#ab46545) and nitrotyrosine (#ab125106) were purchased from Abcam (Cambridge, MA, USA), and GAPDH (#SC-32233) and β-actin (#SC-47778) were purchased from Santa Cruz Biotechnology (Santa Cruz, CA, USA). The Alexa Fluor 488- and 594-conjugated secondary antibodies were purchased from Invitrogen (Carlsbad, CA, USA).

### Smoking mouse and cigarette smoke extract

Smoking mice were performed according to previous study [22]. In brief, six-week-old male BALB/c mice obtained from National Laboratory Animal Center (Taipei, Taiwan) were maintained on standard laboratory food and water ad libitum in the animal centers at the Taipei Medical University. All animal studies were performed in accordance with the rules of the Animal Protection Act of Taiwan, and the animal use protocols were approved by the Laboratory Animal Care and Use Committee of Taipei Medical University, Taiwan (LAC-2014-0168). Mice were whole-body exposed to CS by a CS chamber with the main-stream smoke from the combustion of 12 reference cigarettes (3R4F; Tobacco and Health Research Institute, KY, USA) for a period of approximately 50 min/day, 5 days/week, for 12 weeks. Control mice were exposed to CS-free HEPA-filtered air. Cigarette smoke extract (CSE) was prepared according to previous study with the slight modification [23]. Two filtered cigarettes (Marlboro MX, tar: 10 mg, nicotine content: 0.8 mg; Philip Morris, Switzerland) was imported a vessel containing 6 ml medium by a vacuum pump. Freshly prepared CSE were next sterilized by passing through a 0.22-µm filter and recognized as 100% concentrations of CSE.

### Clinical samples

Clinal samples including lung tissue, PBMC, and BALF were obtained from healthy non-smokers, a healthy smoker, and COPD patients. Patients with COPD were diagnosed and graded according to the guidelines of the Global Initiative for Obstructive Lung Disease [24], and the details of COPD patients were summarized in our previous study [22]. The clinical study protocol was approved by Taipei Medical University-Joint Institutional Review Board (TMU-JIRB No. 201310027) and performed in accordance with the relevant guidelines and regulations. The lung tissues were obtained from COPD patients with lung surgery for the peripheral lung tumor removal, whereas normal control tissues were derived from the noninvolved lung segments of the tumor lesion from non-COPD patients.

### Generation of itch ShRNA

To stably express a lentivirus-based short hairpin RNA (shRNA) targeting *itch*, TRCN0000026925 (5′-CCACCTGAAATACTTTCGTTT-3′), TRCN0000026908 (5′- CCCTACGAGTAAATTATGTTT-3′), and TRCN0000026914 (5′-GCGAAGGAATTAGAGGTTCTT-3′) were obtained from the National RNAi Core Facility (Institute of Molecular Biology/Genomic Research Center, Academia Sinica, Taiwan) followed by the preparation of lentiviral mouse Itch shRNAs from the RNAi Core of Research Center of Clinical Medicine (National Cheng Kung University Hospital, Taiwan). TRCN0000072247 (5′- GAATCGTCGTATGCAGTGAAA-3′) was used as the control luciferase shRNA (shLuc). RAW 264.7 cells were subsequently infected with an appropriate MOI for 24 h followed by puromycin (Calbiochem, San Diego, CA) selection. The protein expression was then measured by western blot analysis.

### Western blot analysis

The total proteins were extracted from RAW264.7 cells, BALF pellets, PBMCs, and mouse lung tissue homogenates using a Triton X-100 based lysis buffer with a protease inhibitor mix and a phosphatase-inhibitors cocktail I followed by the centrifugation at 13,300 rpm for 10 min. Proteins were then resolved using SDS-PAGE and transferred to polyvinylidene difluoride membrane (Millipore Corporation, Billerica, MA, USA). After blocking, the membranes were probed with the indicated primary antibodies (1:1000 dilution) followed by secondary antibodies (1:5000 dilution), and developed by an ECL Western blot detection kit (Pierce Chemical, Rockford, IL, USA) according to the manufacturer’s instructions. All immunoblotting studies were performed in at least two independent experiments, and the relative band intensity on the blots was quantified using Image J software (NIH, Bethesda, MD, USA).

### Histological analysis and immunostaining

Lung tissues obtained from patients and smoking mice were fixed in 10% neutral-buffered formalin, embedded in paraffin wax, and sliced. For histopathology, Sect. (5 μm) were stained with hematoxylin and eosin (H&E). For immunohistochemical analysis, lung tissues were deparaffinized and rehydrated with xylene and different concentrations of ethanol. After permeabilized with 0.1% Triton X-100 in PBS and incubated with 3% hydrogen peroxide, sections were blocked (1% BSA + 0.1% azide in PBS) and stained with specific antibodies against phospho-NF-κB (Ser536), TXNIP, CD11b (clone M1/70, BioLegend San Diego, CA, USA), hydroxynonenal, nitrotyrosine, and Itch followed by HRP- or Alexa Fluor 488- or Alexa Fluor 594-conjugated secondary antibody staining. RAW264.7 cells were fixed by 4% paraformaldehyde in PBS and permeabilized. Fixed cells were then stained with specific antibodies against NF-κB and TXNIP followed by secondary antibody staining. Hematoxylin and DAPI (5 µg/ml) were used for nuclear staining. Images were captured by using fluorescence microscopy (EVOS M5000, Thermo Fisher Scientific, Waltham, MA, USA) and LEICA TCS SP5 laser scanning confocal microscopy system (Leica, Heidelberg, Germany).

### NO and ROS detection

Nitrite accumulation in the cell culture medium was used as an indicator of NO production, detected by the Griess reaction. Briefly, supernatants were mixed with an equal volume of Griess reagent (1% sulfanilamide, 0.1% naphthylethylenediamine dihydrochloride, and 2.5% H3PO4) and incubated for 10 min at room temperature. The relative optical density (OD) of nitrite was measured at 540 nm, and the concentration was evaluated by using sodium nitrite as a standard. For ROS detection, cells were treated with or without TPA and then co-incubated with 20 µM carboxymethyl-H2-dichlorofluorescein diacetate (CM-H2DCFDA, C6827, Thermo Scientific) fluoroprobe for 30 min at 37 °C in the dark. After washing, cells were collected and analyzed using flow cytometry (FACSCalibur, BD Biosciences, San Jose, CA, USA) with the excitation at 488 nm. The emission was detected with the FL-1 channel followed by CellQuest Pro 4.0.2 software (BD Biosciences) analysis, and quantification was performed using FlowJo software (Tree star, Inc., Ashland, Or, USA). The percentages of ROS-positive cells each group were shown.

### NF-κB reporter assay

Expression vectors of pNF-κB-Luc plasmid (Stratagene, La Jolla, CA) and cytomegalovirus-Renilla luciferase construct (pRL-CMV) (Promega, Madison, WI) were transiently cotransfected into cells for 24 h using lipofectamine reagents (Invitrogen, Carlsbad, CA). The Renilla-derived luciferase reporter plasmid was used for transfection efficiency control. In the presence or absence of inhibitors treated for 1 h, cells were stimulated with H_2_O_2_ for 4 h followed by the detection of firefly luciferase activity using Dual Luciferase^®^ Reporter assay system (Promega, Madison, WI, USA) and the multimode reader (Varioskan Flash, Thermo Scientific) according to the manufacturer’s instructions.

### Statistical analysis

Statistical analyses were performed using Student’s t-test (two groups) or one-way ANOVA (more than two groups) followed by a Tukey’s multiple comparison test with Prism 7.0 (GraphPad). The data are presented as the mean ± standard error of the mean (SEM) from three independent experiments. Statistical significance was set at *p < 0.05, **p < 0.01, and ***p < 0.001.

## Results

### Cigarette smoke extract initiates TXNIP reduction and iNOS expression

**Fig. 1:**
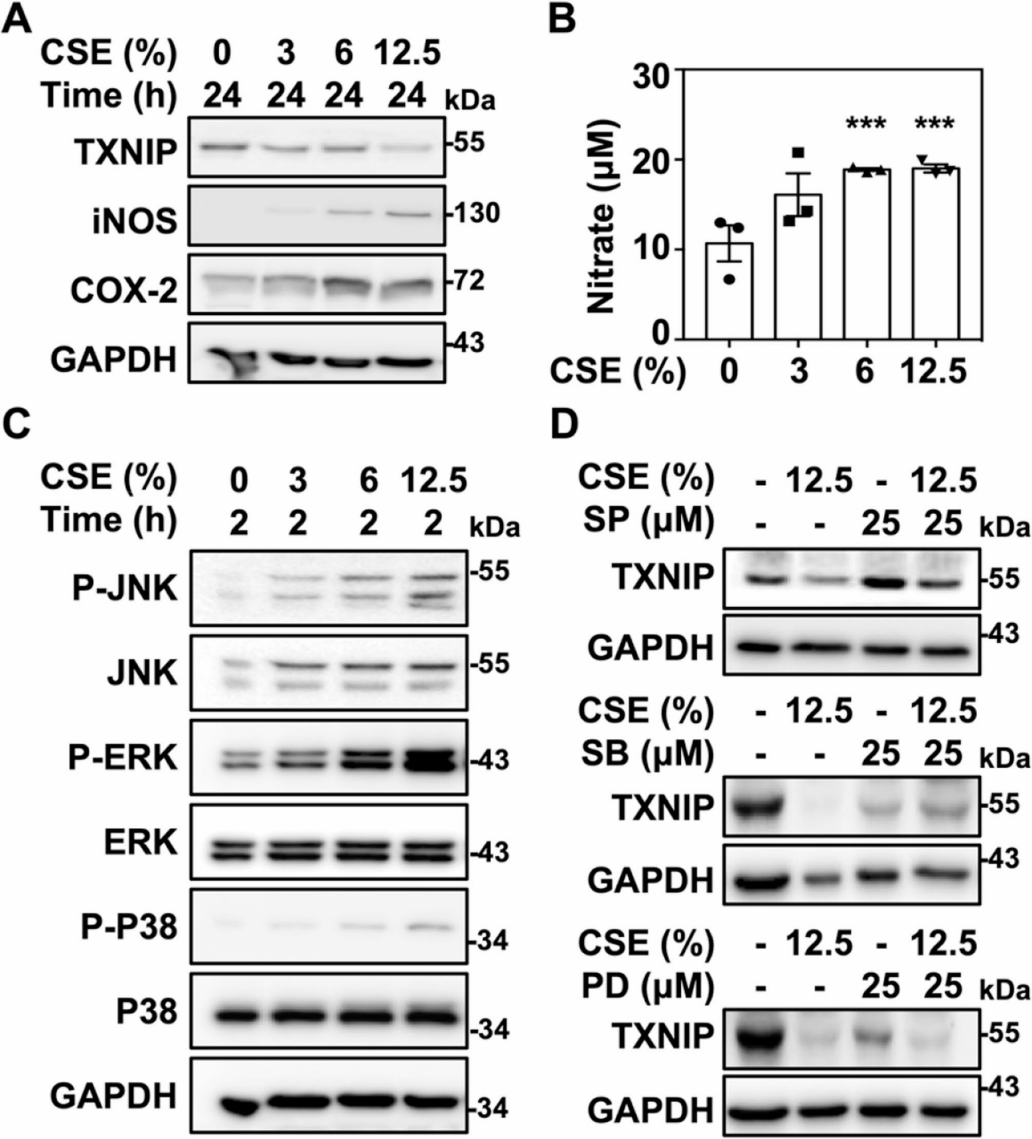
CSE initiates TXNIP downregulation.** A** RAW264.7 cells were treated with indicated dosages of CSE for 24 h, the expressions of TXNIP, iNOS, and COX-2 were detected. GAPDH was used as an internal control. **B** NO release was detected after 24 h CSE exposure. ***p < 0.001 vs. the untreated group. **C** The expression of phospho-JNK, JNK, phospho-ERK, ERK, phospho-p38, and p38 after CSE exposure were detected by western blot analysis. GAPDH was used as an internal control. **D** Cells were pretreated with or without SP600125 (SP), SB203580 (SB), and PD98059 (PD) for 1 h followed by CSE exposure for 24 h. The expression of TXNIP and GAPDH was detected. Protein molecular weights are indicated as kilodaltons (kDa)

TXNIP is involved in a wide variety of cellular processes. Overexpression of TXNIP enables cell apoptosis while TXNIP deficiency causes tumorigenesis and the exacerbation of endotoxic shock [25–27]. To investigate whether TXNIP participates in CS-mediated inflammation, TXNIP expression was measured in CSE stimulation. Freshly prepared CSE markedly attenuated TXNIP expression, accompanied by the induction of inducible nitric oxide synthase (iNOS) and cyclooxygenase (COX)−2 in murine RAW264.7 cells **(**Fig. 1A**)**. CSE significantly induced nitric oxide (NO) production in a dose-dependent manner **(**Fig. 1B**)**, while showing no significant induction of cytotoxicity. Similar to previous reports [28], CSE could effectively induce JNK, p38 MAPK, and ERK phosphorylation in RAW264.7 cells **(**Fig. 1C**)**. Moreover, the presence of JNK and p38 MAPK inhibitors, SP600125 and SB203580, markedly alleviated CSE-caused TXNIP reduction respectively **(**Fig. 1D**)**. TXNIP has been linked to regulate stress-activated apoptosis and inflammation through its cellular expression and distribution [9]. Interestingly, TXNIP was distinctly suppressed instead of being induced in coordination with the elevation of inflammatory mediators in CSE stimulation. This might suggest a novel role for TXNIP in CS-regulated inflammation.

### Decreased TXNIP expression in the lungs of smoking mice

**Fig. 2:**
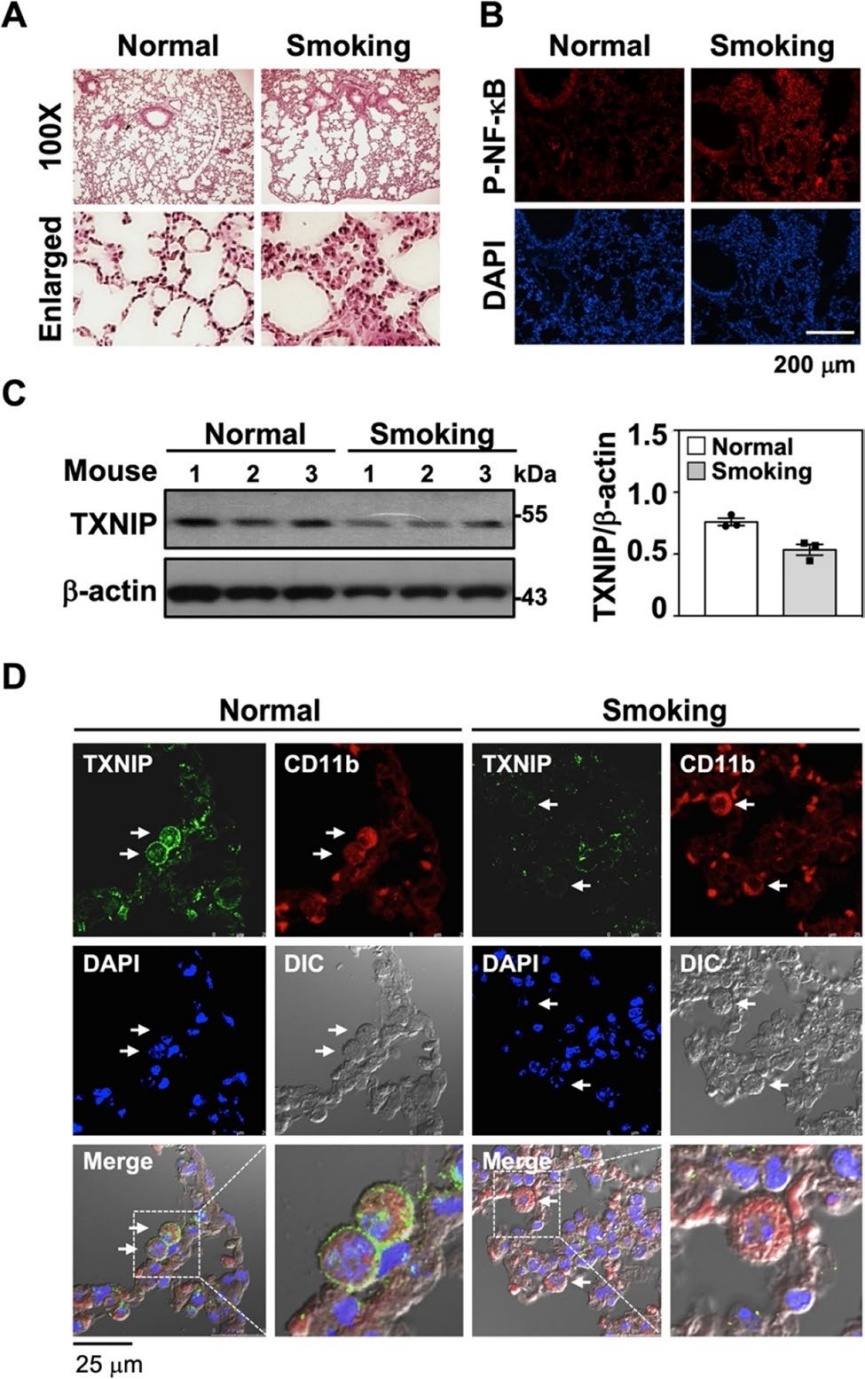
Smoking mice present lung inflammation, NF-κB activation, and TXNIP downregulation.** A** The hematoxylin and eosin (H&E) staining of lung sections from mice with or without a 12-week time course of smoking exposure were shown. Microphotographs are shown at 100× magnification. **B** Immunofluorescence staining was performed to detect the expression of phospho-NF-κB p65 (Ser536) in lung tissues of normal and smoking mice using specific antibodies followed by Alexa594-conjugated secondary antibodies staining (*Red*). DAPI staining was used to determine the nuclei. Scale bar is 200 μm. **C** TXNIP expression in total protein isolated from normal (*n* = 3) and smoking mouse lung tissues (*n* = 3) were measured by immunoblotting. β-actin was used as an internal control, and the ratios of TXNIP to β-actin were shown as the means ± SEM. **D** Lung tissues obtained from normal and smoking mice were stained with TXNIP antibodies followed by Alexa488-conjugated secondary and CD11b-Alexa594-conjugated antibodies. DAPI was used for nuclear staining. The immunofluorescence and differential interference contrast (DIC) images were determined by confocal microscope and shown. Scale bar is 25 μm

Down-regulated TXNIP expression was observed in CSE-stimulated murine macrophage RAW264.7 cells. Subsequently, changes in pulmonary TXNIP expression in smoking mice were investigated. Following 12 weeks of CS exposure, the mice exhibited significant immune cell infiltration in the lungs and abnormal enlargement of airspaces **(**Fig. 2A**)**. NF-κB activation was determined in the lungs of smoking mice, revealing elevated phosphorylation of p65 NF-κB at serine 536 **(**Fig. 2B**)**. This indicated that CS exposure could effectively trigger NF-κB-regulated inflammation. Further assessments of TXNIP expression revealed significant decreases in TXNIP levels in the lung tissues of smoking mice compared to normal mice **(**Fig. 2C**)**. Additionally, TXNIP expression was intensely suppressed in lung macrophages expressing CD11b **(**Fig. 2D**)**. Consistent with previous results, the downregulation of TXNIP might potentially accompany NF-κB activation in macrophages, leading to pulmonary inflammation induced by CS stimulation.

### Oxidative stress-regulated proteasomal degradation of TXNIP

**Fig. 3:**
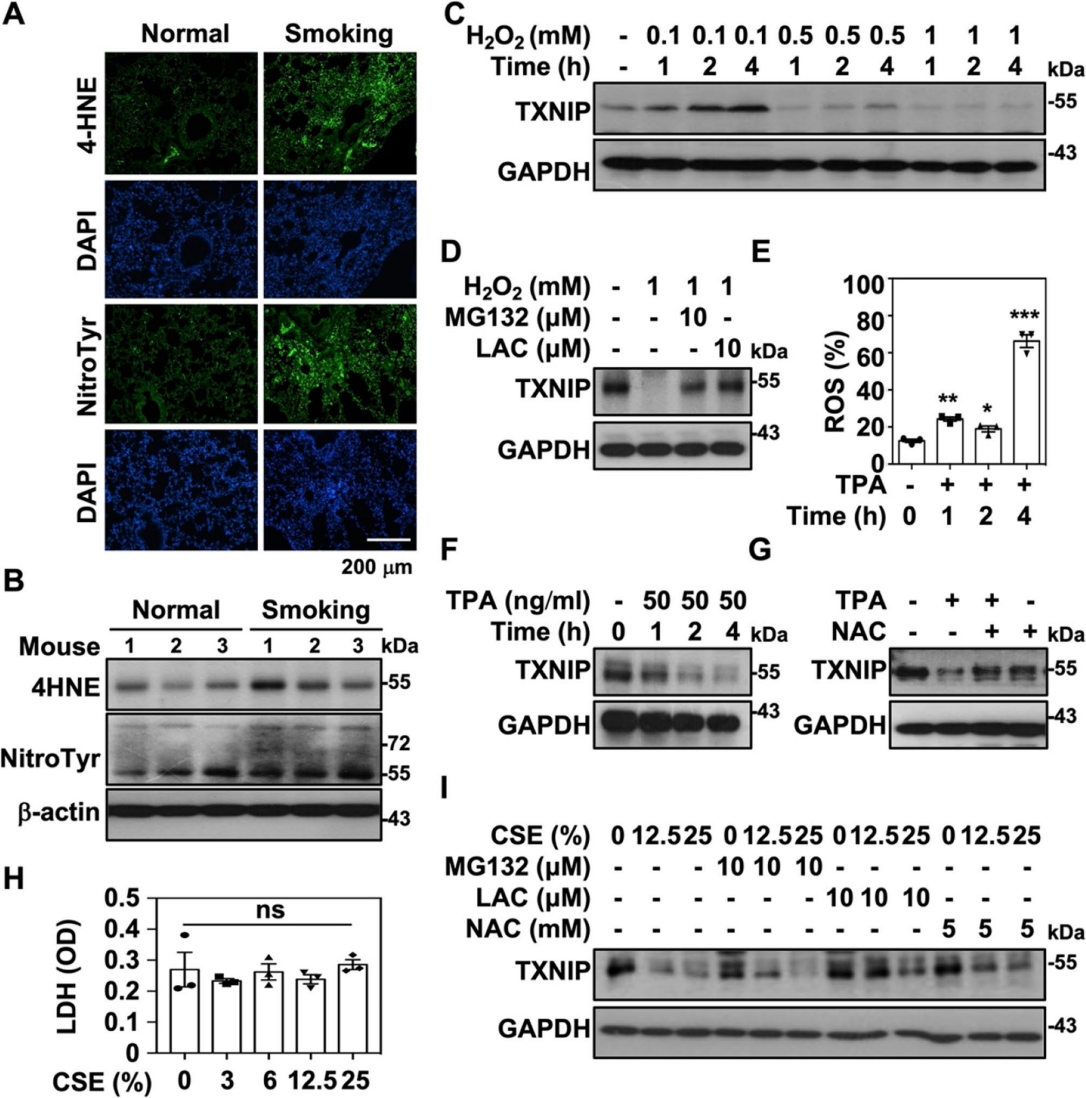
Oxidative stress mediates TXNIP proteasomal degradation.** A** Immunofluorescence staining was performed to detect the expression of 4-hydroxynonenal (4-HNE) and nitrotyrosine (NitroTyr) in lung tissues of normal and smoking mice respectively using specific antibodies followed by Alexa488-conjugated secondary antibodies staining (*Green*). DAPI staining was used to determine the nuclei. Scale bar is 200 μm. **B** The expression of 4-HNE and NitroTyr in total protein isolated from normal (*n* = 3) and smoking mouse lung tissues (*n* = 3) were measured, and β-actin was used as an internal control. **C)** RAW264.7 cells were treated with various dosages of H_2_O_2_ for indicated time points, and the protein expression of TXNIP and GAPDH were detected. **D** In the presence of MG132 (10 µM) and lactacystin (10 µM), cells were treated with H_2_O_2_ (1 mM) for 2 h followed by the determination of TXNIP and GAPDH expression. **E** RAW264.7 cells were treated with TPA (50 ng/ml) for indicated time points followed by the ROS detection. The relative percentages of ROS production were measured, and shown as the means ± SEM from triplicate cultures. *p < 0.05, **p < 0.01, ***p < 0.001. **F** TXNIP expression in TPA (50 ng/ml)-treated RAW264.7 cells was detected at indicated time points. **G** Cells were pretreated with or without NAC (5 mM) for 1 h followed by TPA (50 ng/ml) stimulation for 2 h. TXNIP expression was detected, and GAPDH was used as an internal control. **H** RAW264.7 cells were exposed to indicated dosages of CSE for 24 h followed by the measurements of LDH release. **I** In the presence or absence of MG132 (10 µM), lactacystin (10 µM), and NAC (5 mM), cells were exposed to indicated dosages of CSE for 24 h followed by detecting TXNIP expression. GAPDH was used as an internal control. Protein molecular weights are indicated as kilodaltons (kDa)

CS represents one of the most significant exogenous oxidants, containing abundant ROS, hydrogen peroxide (H_2_O_2_), and NO that contribute to oxidative stress-mediated inflammation [29]. Given the marked downregulation of TXNIP in the lung tissue of smoking mice, we subsequently explored whether TXNIP expression is regulated by oxidative stress. The distinct elevations of a sensitive marker of oxidative damage and lipid peroxidation, 4-hydroxynonenal (4-HNE), and a versatile oxidative stress biomarker, nitrotyrosine (NitroTyo), were detected in smoking murine lung tissues by immunofluorescence staining **(**Fig. 3A**)** and western blot analysis **(**Fig. 3B**)**. These results suggested the potential coordination between TXNIP downregulation and oxidative stress. Exogeneous treatment of H_2_O_2_ showed a dose-dependent TXNIP downregulation in RAW264.7 cells **(**Fig. 3C**)**. The presence of proteasome inhibitors, MG132 and lactacystin (LAC), distinctly reversed H_2_O_2_-caused TXNIP downregulation **(**Fig. 3D**)**, which suggested that oxidative stress might initiate TXNIP undergoing proteasomal degradation. Furthermore, the endogenous ROS induced by 12-O-tetradecanoylphorbol-13-acetate (TPA) **(**Fig. 3E**)** similarly induced TXNIP degradation in RAW264.7 cells **(**Fig. 3F**)**, while the ROS inhibitor, N-acetylcysteine (NAC), markedly attenuated TPA-induced TXNIP degradation **(**Fig. 3G**)**. In addition, the various concentrates of freshly prepared CSE we used showed no significant cytotoxicity in RAW264.7 cells **(**Fig. 3H**)**. Notably, the presence of MG132, LAC, and NAC similarly attenuated the CSE-induced TXNIP degradation **(**Fig. 3I**)**. Therefore, cigarette smoking might induce oxidative stress-regulated TXNIP proteasomal degradation.

### ROS mediates MAPK-regulated TXNIP degradation and NF-κB activation

**Fig. 4:**
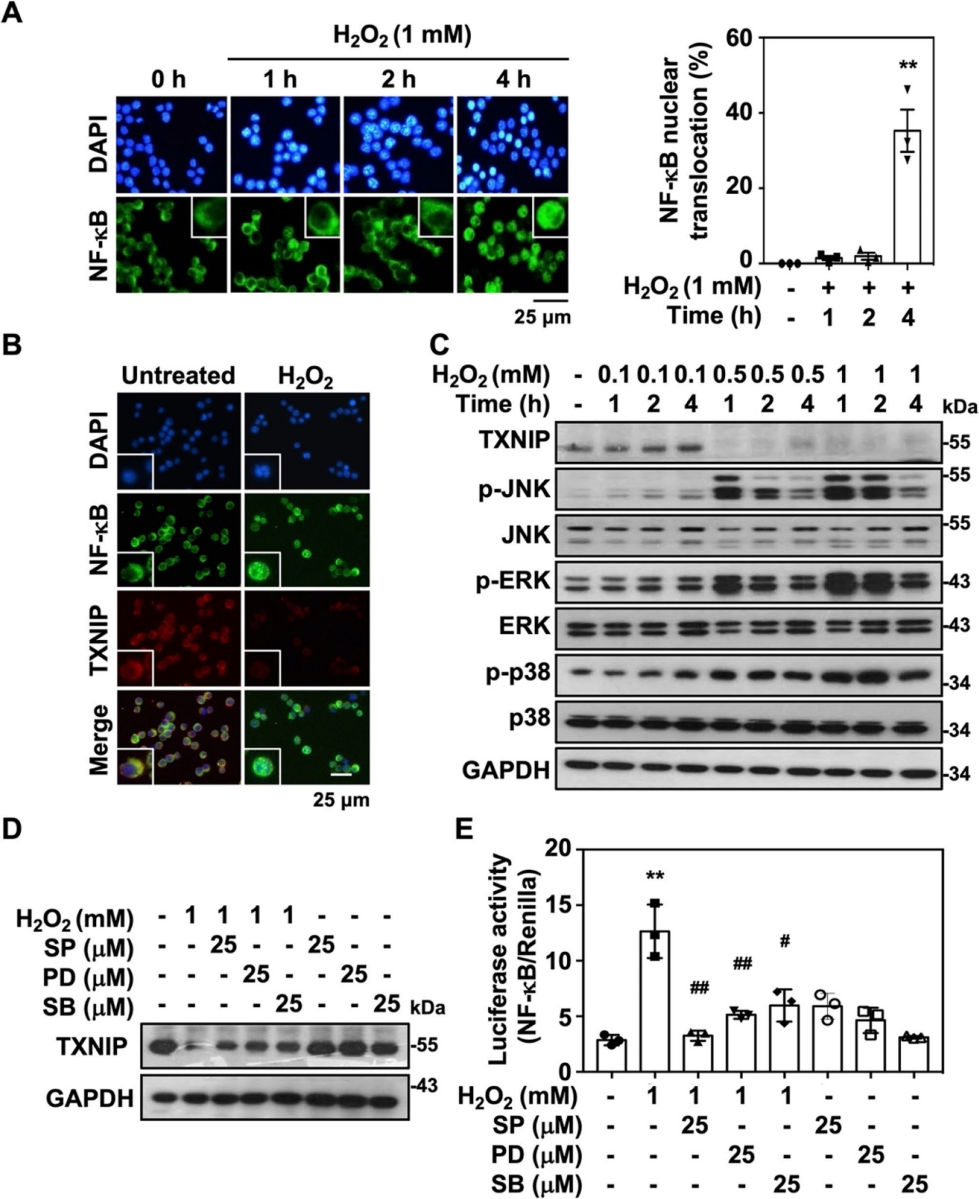
MAP-Kinases regulate oxidative stress-mediated TXNIP degradation and NF-κB activation. **A** The nuclear translocation of NF-κB in H_2_O_2_-treated RAW264.7 cells were assayed at indicated time points using a specific antibody against NF-κB (*Green*) followed by fluorescence microscopic observation. DAPI was used as a nuclear staining, and scale bar was shown. Quantitative measurements of NF-κB nuclear translocation (percentages based on more than 150 cells in total) were performed and shown as the means ± SEM of triplicate cultures. **p < 0.01. **B** RAW264.7 cells were treated with H_2_O_2_ (1 mM) for 4 h and subsequently stained with TXNIP (*Red*) and NF-κB (*Green*) respectively. DAPI was used as a nuclear staining, and scale bar was shown. **C** RAW264.7 cells were treated with different dosages of H_2_O_2_ for indicated time points, the expressions of TXNIP, phospho-JNK (Thr183/Tyr185), JNK, phospho-ERK (Thr202/Tyr204), ERK, phospho-p38 (Thr180/Tyr182), and p38 were determined. GAPDH was used as an internal control. **D** RAW264.7 cells were preincubated with 25 µM of SP600125 (SP), PD98059 (PD), and SB 253580 (SB) for 1 h followed by 1 mM of H_2_O_2_ treatment for 2 h. The expression of TXNIP and GAPDH were measured. Protein molecular weights are indicated as kilodaltons (kDa). **E** The luciferase reporter assay of NF-κB was measured after 1 mM of H_2_O_2_ treatment for 4 h in the presence or absence of inhibitors. Triplicate cultures were performed and shown as the means ± SEM. **p < 0.01 as compared to control; ^##^ *p* < 0.01 and ^#^*p* < 0.05 as compared to H_2_O_2_ groups

NF-κB is a critical transcriptional factor of the inflammatory process, while H_2_O_2_ has been revealed as a fine-tuning regulator of NF-κB-dependent inflammation [30]. In H_2_O_2_-stimulated RAW264.7 cells, the nuclear translocation of NF-κB was significantly increased **(**Fig. 4A**)**. Furthermore, NF-κB nuclear translocation occurred concurrently with TXNIP degradation in the cells **(**Fig. 4B**)**. H_2_O_2_ treatment effectively induced TXNIP degradation, which was in associated with the phosphorylation of JNK, p38 MAPK, and ERK **(**Fig. 4C**)**. Pharmacological inhibition of MAPKs using specific inhibitors, SP600125, PD98059, and SB203580, which inhibited JNK, ERK, and p38 MAPK, respectively, suppressed H2O2-induced TXNIP degradation **(**Fig. 4D**)**, as well as subsequent NF-κB activation **(**Fig. 4E**)**. Accordingly, ROS-induced NF-κB activation may be regulated by MAPK-mediated TXNIP degradation.

### Itch expression modulates TXNIP-mediated NF-κB activation

**Fig. 5:**
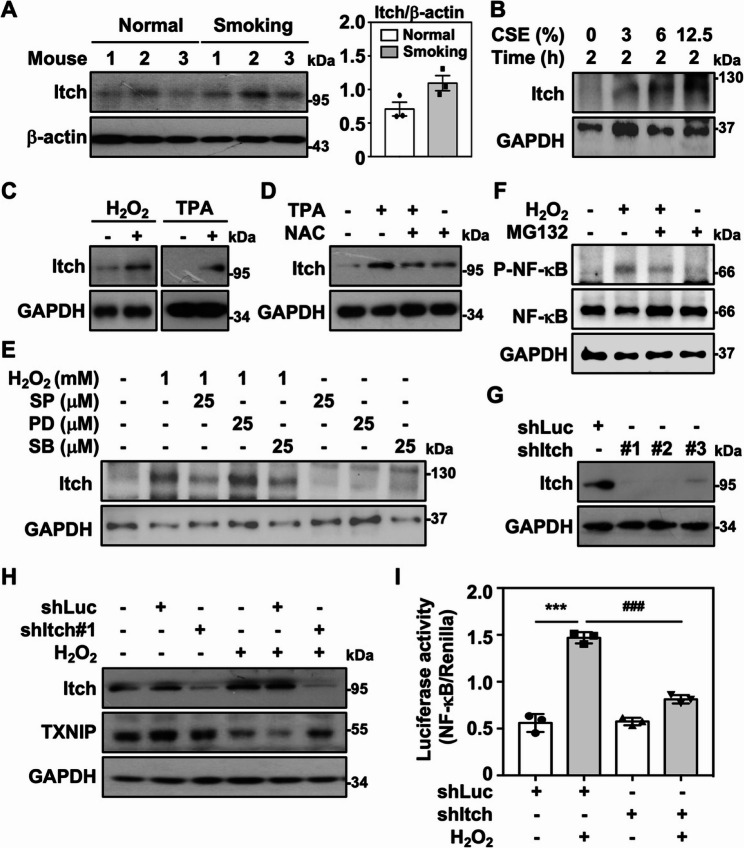
Itch expression modulates TXNIP-mediated NF-κB activation. **A** Itch expression in total protein isolated from normal (*n* = 3) and smoking mouse lung tissues (*n* = 3) were measured by immunoblotting. β-actin was used as an internal control, and the ratios of Itch to β-actin were shown as the means ± SEM. **B** RAW264.7 cells were stimulated with CSE for 2 h, and Itch expression was subsequently measured. GAPDH was used as an internal control. **C** The expression of Itch and GAPDH were measured in RAW 264.7 cells treated with H_2_O_2_ (1 mM) and TPA (50 ng/ml) for 2 h. **D** In the presence of 5 mM NAC, the expression of Itch and GAPDH were detected in cells treated with TPA (50 ng/ml) for 2 h. **E** Cells were pretreated with or without SP600125 (SP), SB203580 (SB), and PD98059 (PD) for 1 h followed by H_2_O_2_ treatment for 2 h. The expression of Itch and GAPDH was detected. **F** In the presence or absence of MG132 (10 µM), and the expression of phospho-NF-κB (Ser536), NF-κB, and GAPDH were detected in cells treated with H_2_O_2_ (1 mM) for 4 h. **G** RAW264.7 cells were expressed with shRNA targeting luciferase (shLuc) and shRNA targeting Itch (shItch#1, shItch#2, and shItch#3), and the expression of Itch and GAPDH were subsequently confirmed by immunoblotting. **H** Cells expressed shLuc and shItch#1 were treated with or without H_2_O_2_ for 2 h. The expression of Itch, TXNIP, and GAPDH were detected. Protein molecular weights are indicated as kilodaltons (kDa). **I** The luciferase reporter assay of NF-κB was measured in shLuc- and shItch-cells stimulated with H_2_O_2_ (1 mM) for 4 h. The quantitative data are presented as the means ± SEM from triplicate cultures. ***p < 0.001 as compared to untreated. ^###^*p* < 0.001 as compared to the shLuc-group

TXNIP has been shown to interact with the ubiquitin E3 ligase Itch through a conserved PPXY motif in the C terminus of TXNIP and undergoes degradation [31]. Itch expression is initially slightly increased by ROS stimulation, but subsequently decreases following TXNIP suppression in cardiomyocytes [32]. Similarly, we observed a slight increase in Itch expression in lung tissue extracted from smoking mice **(**Fig. 5A**)**. In vitro, CSE exposure induced a concentration-dependent increase in Itch expression in RAW264.7 cells **(**Fig. 5B**)**. Moreover, H₂O₂ and TPA stimulated Itch expression in RAW264.7 cells **(**Fig. 5C**)**, while TPA-elevated Itch expression was partly inhibited by the presence of NAC **(**Fig. 5D**)**. Thus, ROS accumulation upon CS exposure may potentiate Itch induction. Previously, MAPKs were shown to act upstream in modulating TXNIP degradation. We therefore examined whether ROS-mediated Itch induction is also regulated by MAPK signaling. In the presence of the MAPK inhibitors SP600125 and SB203580, H₂O₂-stimulated Itch expression was partly inhibited **(**Fig. 5E**)**. In addition, MG132 stabilized TXNIP, which in turn attenuated H₂O₂-stimulated NF-κB phosphorylation **(**Fig. 5F**)**, suggesting that TXNIP expression partially influences ROS-stimulated NF-κB activation. Since TXNIP degradation may occur in accordance with Itch expression, we further explored whether the specific knockdown of Itch might affect ROS-induced TXNIP degradation. By using RNA interference, Itch expression was specifically silenced in RAW264.7 cells **(**Fig. 5G**)**. H_2_O_2_-induced TXNIP degradation was markedly reversed in Itch knockdown RAW264.7 cells compared to the controls **(**Fig. 5H**)**. Moreover, H_2_O_2_-mediated NF-κB activation was significantly attenuated in Itch knockdown cells accompanying by the stabilization of TXNIP **(**Fig. 5I**)**. Therefore, ROS-mediated upregulation of Itch could potentially lead to TXNIP degradation, resulting in subsequent NF-κB activation in macrophages.

### Decreased TXNIP levels in COPD patients

**Fig. 6:**
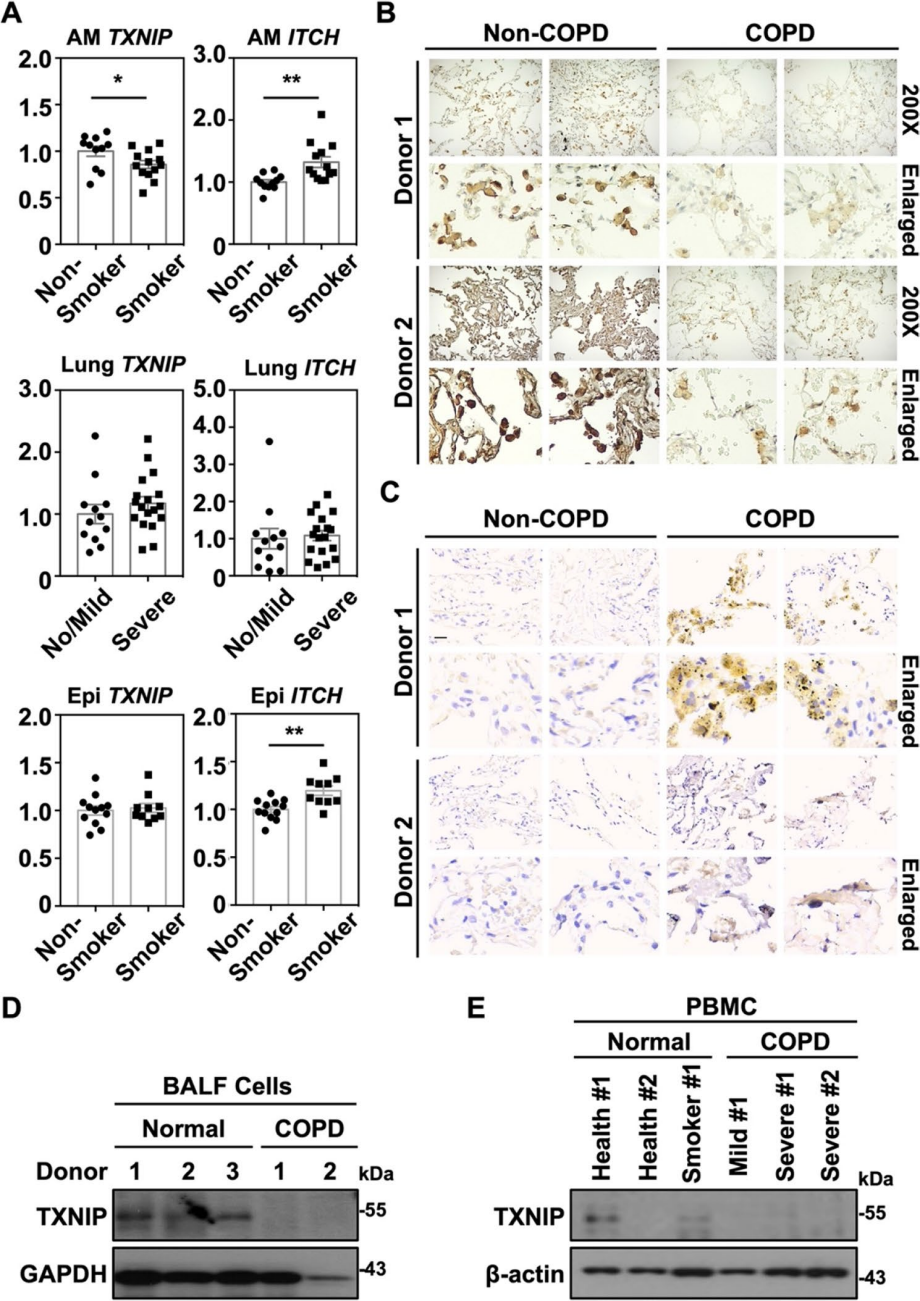
Decreased expression of TXNIP in COPD patients.** A** Gene expression of *TXNIP* (GDS3496/201010_s_at) and *ITCH* (GDS3496/217094_s_at) in alveolar macrophages of cigarette smokers obtained from the public GEO profiles was assayed. Gene expression of *TXNIP* (GDS737/201010_s_at) and *ITCH* (GDS737/209743_s_at) in smoker lung tissues with no/mild and severe emphysema obtained from the public GEO profiles was assayed. Gene expression of *TXNIP* (GDS2468/201010_s_at) and *ITCH* (GDS2468/217094_s_at) in small airway epithelium of cigarette smokers obtained from the public GEO profiles was assayed. The relative fold changes were shown as means ± SEM. *p < 0.05, **p < 0.01. Immunohistochemical staining was performed using specific antibodies to detect the expression of TXNIP **(B)** and Itch **(C)** in the fixed lung tissue sections obtained from COPD patients. Normal controls were obtained from the noninvolved lung sections of the tumor lesion. Microphotographs are shown at 200× and enlarged magnification. The scale bar is shown as 30 μm. **D** Bronchoalveolar lavage fluid (BALF) cells were collected from donors with or without COPD, and subsequently performed the immunoblotting to measure TXNIP expression. GAPDH was used as an internal control. **E** Peripheral blood mononuclear cells (PBMC) were isolated from healthy donors (*n* = 2), smokers (*n* = 1), mild COPD patients (*n* = 1), and severe COPD patients (*n* = 2). TXNIP expression was detected and β-actin was used as an internal control. Protein molecular weights are indicated as kilodaltons (kDa)

TXNIP protein expression exhibits distinct variations in different cells response to ROS stimulation [33, 34], where we observed ROS-mediated TXNIP suppression in lung tissues of smoking mice as well as in murine macrophages. Exploration of gene profiles obtained from DataSet Record GDS3496 and GDS737 on Gene Expression Omnibus (GEO) at the National Center for Biotechnology Information (NCBI) revealed that the significant relative lower expression of *TXNIP* and higher expression of *ITCH* were shown in alveolar macrophages of cigarette smokers (*n* = 13) compared to nonsmokers (*n* = 11), while no significant changes of *TXNIP* and *ITCH* were found in lung tissues from smokers with severe emphysema (*n* = 18) compared to no or mild emphysema (*n* = 12) **(**Fig. 6A**)**. Interestingly, analysis of gene profiles from DataSet Record GDS2468 also revealed significantly higher expression of *ITCH* in the small airway epithelium of cigarette smokers (*n* = 10) compared with non-smokers (*n* = 12), whereas *TXNIP* expression showed no significant difference **(**Fig. 6A**)**. To further assess the expression of TXNIP and Itch in oxidative stress-associated lung diseases, we collected lung tissues from non-COPD and COPD patients undergoing lung surgery for peripheral lung tumor removal. TXNIP expression was markedly reduced in COPD lung tissues compared with non-COPD patients **(**Fig. 6B**)**, whereas Itch expression was elevated **(**Fig. 6C**)**. Similarly, decreased TXNIP expression was observed in BALF cells from COPD patients **(**Fig. 6D**)**. Furthermore, PBMCs obtained from healthy non-smokers, healthy smokers, and COPD patients revealed significantly greater inhibition of TXNIP in mild and severe COPD patients compared to healthy donors **(**Fig. 6E**)**. Accordingly, consistent CS exposure or strong oxidant stimulation can potentially enhance Itch expression, which in turn promotes TXNIP proteasomal degradation and downstream NF-κB activation.

## Discussion

**Fig. 7:**
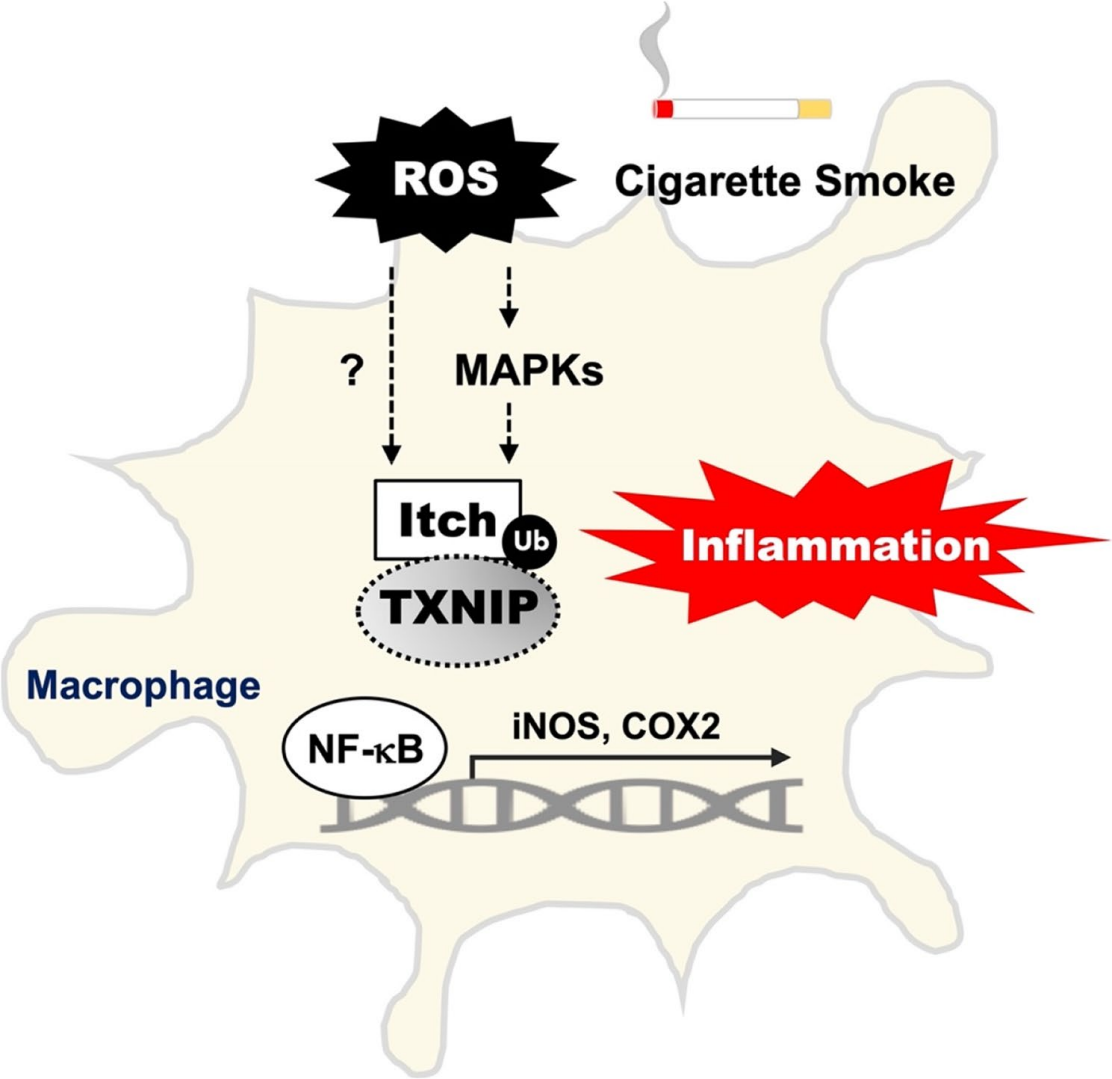
A schematic model of TXNIP downregulation promoting NF-κB-mediated inflammation in response to CS stimulation. CS contains thousands of chemicals, including abundant free radicals. Oxidative stress initiates the activation of ERK, JNK, and p38, thereby leading to HECT-type E3 ubiquitin ligase Itch-regulated TXNIP degradation. The downregulation of TXNIP may effectively enable NF-κB activation and the subsequent induction of proinflammatory iNOS/NO and COX-2. Therefore, sustained exposure to CS and oxidative stress may potentially cause TXNIP downregulation and chronic inflammatory responses, potentiating the progression of COPD

The burden of CS-induced oxidative stress, cellular damage, and inflammation are major contributors to COPD pathogenesis [35]. TXNIP possesses pro-oxidative and pro-inflammatory characteristics, leading to the initiation or exacerbation of inflammation and cellular damages in various disease progressions [36–38]. TXNIP induction has also been reported to play a role in the development of inflammation-related lung disorders, including acute lung injury, allergic exacerbation, lung fibrosis, and COPD exacerbation [16–20, 38]. In this study, we demonstrate that TXNIP reduction is associated with CSE-induced inflammation in RAW264.7 cells and with lung inflammation in smoking mice. CS-induced oxidative stress activates MAPK-regulated proteasomal degradation of TXNIP, leading to NF-κB activation. Notably, upon CS exposure and oxidative stimulation, the expression of Itch, the upstream E3 ubiquitin ligase of TXNIP, was increased. Inhibition of Itch significantly attenuated TXNIP degradation and NF-κB activation. Moreover, higher *ITCH* expression was detected in alveolar macrophages and small airway epithelium of cigarette smokers compared with non-smokers, whereas *TXNIP* expression was lower. In COPD patients, Itch was upregulated, while TXNIP was downregulated in lung tissues, BALF cells, and PBMCs. Thus, CS-induced oxidative stress appears to promote MAPK-regulated Itch expression and TXNIP degradation, leading to NF-κB activation and subsequent inflammation **(**Fig. 7**)**. Furthermore, the increased Itch and decreased TXNIP levels observed in COPD patients suggest their potential involvement in regulating disease progression.

TXNIP expression can be triggered by multiple stimuli, including hyperglycemia, ischemia-reperfusion injury, hypoxia, ER stress, and ROS. Under stress conditions, increased TXNIP may either promote apoptosis via apoptotic signal-regulated kinase 1-mediated mitochondrial pathways through TRX-dependent signaling [25, 37], or regulate NLRP3 inflammasome activation in a redox-independent manner [13, 36]. In copper oxide nanoparticle-mediated COPD exacerbation, the TXNIP-NLRP3 signaling pathway has also been shown to play a critical role in initiating inflammatory responses in the respiratory tract [38]. Conversely, TXNIP degradation contributes to tumor necrosis factor-α-stimulated NF-κB activation, while TXNIP deficiency exacerbates lipopolysaccharide (LPS)-induced endotoxic shock and *E. coli*-mediated mortality through excessive NO production [27, 39]. Moreover, toll-like receptor (TLR) 2- and ROS-mediated rapid TXNIP degradation in macrophages has been shown to accelerate NF-κB activation during *Streptococcus pyogenes* infection [40]. Here, we found that CSE stimulation induced dose-dependent TXNIP degradation with concurrent iNOS/NO and COX-2 induction in macrophages, while MAPK-regulated TXNIP degradation further promoted NF-κB activation. A significant increase in emphysema development has been observed in USP13-deficient mice after mild CS exposure, in which TXNIP reduction was also detected in lung epithelial cells with USP13 inhibition [41]. However, other reports indicate that CSE upregulates TXNIP expression and activates the TXNIP-NLRP3-gasdermin D axis to promote inflammation and pyroptosis of islet β-cells [42, 43]. Thus, the stability and functions of TXNIP in response to CS remain controversial and may appear to be regulated in a cell type-specific manner.

Itch, a HECT-type ubiquitin E3 ligase, can interact with TXNIP and regulate its ubiquitin labeling, promoting proteasomal degradation [31, 32, 44]. Itch has long been respected as a critical suppressor of inflammation, which limits Th2 immunity by regulating T cells, B cells, and macrophages. Itch deficiency presents abnormal Th2-related lung and skin inflammation and spontaneous gastrointestinal tract inflammation in mice, along with immune abnormalities in humans, while Itch overexpression has been observed in several human cancers [45, 46]. The promotion or repression of Itch activity is regulated by the phosphorylation status in response to various stimulations of growth factors, death receptors, DNA damage, and oxidative stress [47–49]. In addition to post-translational modification, ROS-mediated increased expression of Itch has also been reported to trigger the degradation of FLICE-like inhibitory protein [50]. Upon CS and ROS stimulation, we detected increased Itch expression in lung tissues from COPD patients, smoking mice, and RAW264.7 cells, accompanied by TXNIP degradation. Consistently, GEO profiles showed that alveolar macrophages and small airway epithelium from smokers exhibited higher *ITCH* gene expression than those from non-smokers, although *ITCH* expression did not significantly change with emphysema progression. Furthermore, ROS-induced Itch expression contributed to downstream NF-κB activation, suggesting a potential proinflammatory role of Itch in CS exposure.

Chronic inflammation induced by abundant ROS stimulation and MAPKs activation is usually the major cause leading to consistent alveolar macrophage activation and alveolar epithelial cell damage in COPD pathogenesis [5, 6, 51]. Targeting oxidative stress with therapeutic agents has been recommended as an effective approach in treating COPD, and several antioxidants, including NAC, have been applied in clinical trials with some beneficial outcomes [51]. In addition to its well-known function of elevating intracellular glutathione levels [5, 51], we demonstrated here that NAC has the capacity to stabilize the ROS-regulated Itch/TXNIP/NF-κB axis, thereby attenuating inflammation. Meanwhile, targeting MAPK signaling is also speculated as another efficient approach for treating inflammatory lung diseases, with clinical trials showing the therapeutic potential of p38 MAPK inhibitors in improving lung function and reducing exacerbations in COPD patients [52, 53]. Here, we investigated that ROS-initiated Itch/TXNIP/NF-κB axis was partly regulated by the activation of JNK, p38 MAPK, and ERK, while the blockage of MAPKs could potentially attenuate NF-κB activation. Given the heterogeneity of COPD pathogenesis, both antioxidants and MAPK inhibitors exhibit only limited therapeutic potential. Therefore, combining these treatments with other anti-inflammatory medications appears to be a promising future therapeutic approach.

TXNIP expression varies across different respiratory disorders. In lung cancer, TXNIP expression is significantly reduced, and its downregulation promotes tumor proliferation and migration while inhibiting apoptosis in lung cancer cell lines [12]. In allergic airway inflammation and lung fibrosis, elevated TXNIP levels contribute to Th2 immune responses in the airway and to oxidative stress in fibrotic lungs, respectively [19, 20]. Although P2 × 7/caspase-1 activation has been reported to contribute to CS-induced lung inflammation in mice and smokers [21], and copper oxide nanoparticles have been shown to exacerbate inflammatory responses through the TXNIP-NLRP3 signaling pathway in a cigarette smoke condensate-induced COPD mice and in human lung mucoepidermoid carcinoma cells [38]. The role of TXNIP in CS-mediated inflammation remains incompletely understood and may vary across cell types. In this study, we demonstrated for the first time that TXNIP downregulation is associated with CS-induced oxidative stress and inflammation in lung tissues and macrophages. Moreover, the ROS/Itch/TXNIP axis acts upstream of NF-κB activation, potentially providing an additional pathway for NF-κB initiation, which is considered critical in COPD-related inflammation.

However, the very limited sample size of smoking mice and COPD patients in the study provided only partial evidence that the oxidative stress-induced Itch/TXNIP/NF-κB axis occurs in CS-exposed macrophages, and it remains unclear whether this axis also arises in epithelial or endothelial cells, both critical players in airway inflammation. Notably, smokers exhibit higher *ITCH* gene expression in alveolar macrophages and small airway epithelium than non-smokers. The increased Itch may possibly initiate the TXNIP/NF-κB pathway in both macrophages and epithelial cells upon CS exposure. Based on our current and previous data, we have demonstrated that oxidative stress- and TLR-mediated TXNIP degradation regulates NF-κB-driven inflammatory responses in macrophages [40], although whether this represents a cell type-specific response remains uncertain. Myeloid-specific *txnip* transgenic mice may be used in the future to verify this mechanism more precisely. In addition, compared with mainstream studies reporting TXNIP-NLRP3 induction [21, 38], our data indicate that CS exposure significantly induces TXNIP protein degradation in macrophages. How to effectively stabilize TXNIP in order to fine-tune NF-κB activation and modulate inflammatory responses remains an urgent issue, particularly since Itch inhibitors exhibit potential cytotoxicity. Furthermore, because TXNIP degradation can occur through both Itch-dependent and -independent pathways [40], validation of this mechanism is even more challenging. Moreover, as CS contains various substances, including LPS, it remains to be determined whether TLR signaling also promotes the TXNIP/NF-κB pathway during CS exposure. COPD is a complex disease with diverse etiologies, and more detailed investigations into its mechanisms will facilitate future drug development.

## Conclusion

In summary, we have demonstrated that CS-induced oxidative stress triggers MAPK activation and Itch expression, leading to TXNIP degradation in macrophages. Itch-dependent TXNIP proteasomal degradation facilitates NF-κB activation and inflammatory responses, potentially contributing to COPD pathogenesis. Stabilizing TXNIP using a ROS scavenger or Itch-specific knockdown can attenuate NF-κB-mediated inflammation, suggesting the potential therapeutic target for COPD treatment.

## Supplementary Information

Additional file 1. Raw western blot images.

## Data Availability

The datasets used and/or analyzed during the current study are available from the corresponding author on reasonable request.
