# Supplementary material for: Oxidative stress triggers Itch-mediated TXNIP degradation and NF-κB activation promoting chronic obstructive pulmonary disease
Source: Respir Res. 2025 Oct 17;26:286. doi: 10.1186/s12931-025-03369-5 (PMC12534957; doi:10.1186/s12931-025-03369-5)

Supplementary Figure 1

Figure 1A

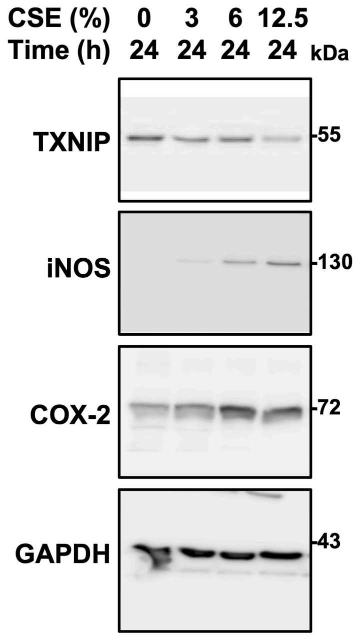

Figure 1C

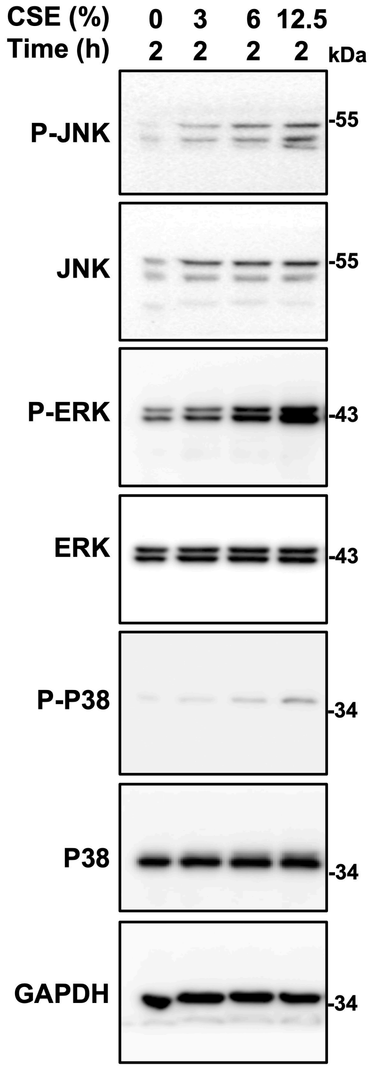

Figure 1D

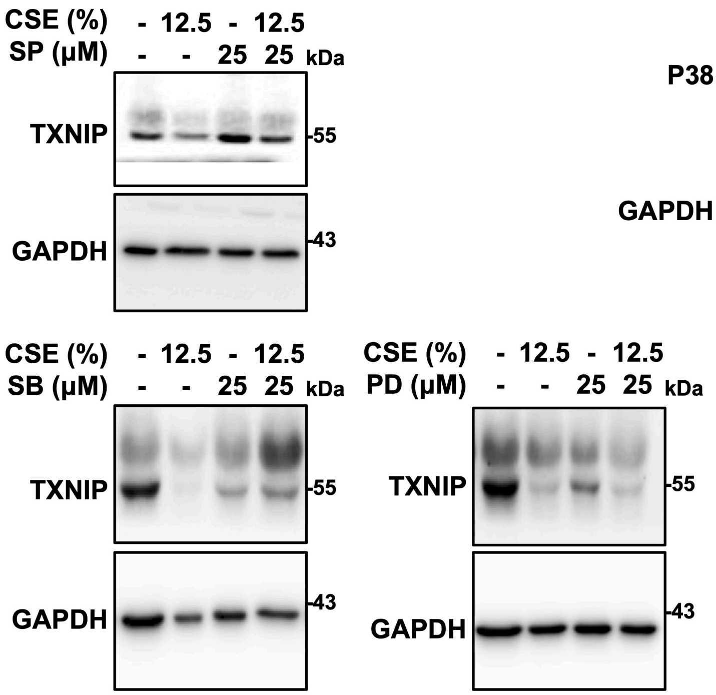

Supplementary Figure 2

Figure 2C

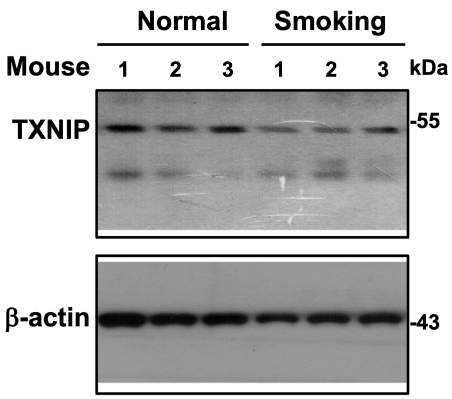

Supplementary Figure 3

Figure 3B

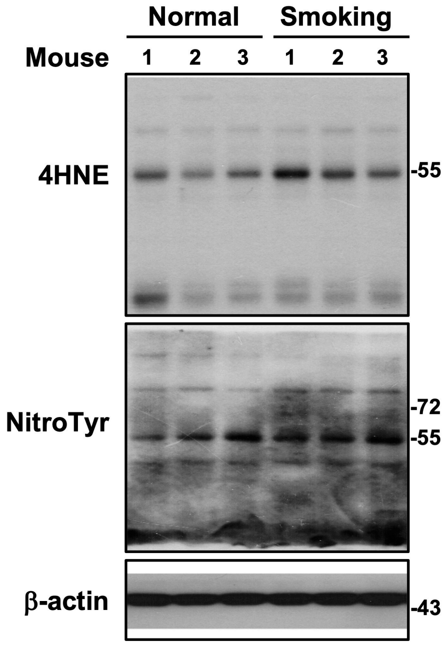

Figure 3C

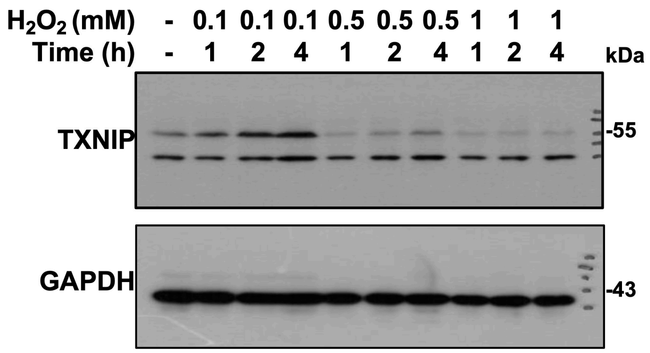

Figure 3D

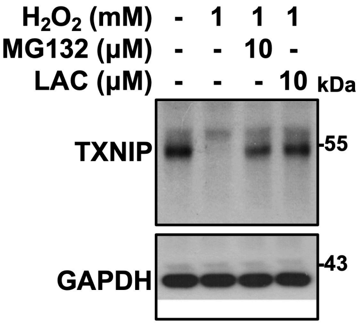

Figure 3F

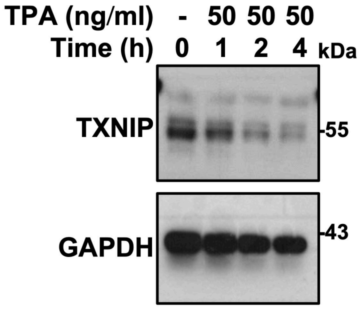

Figure 3G

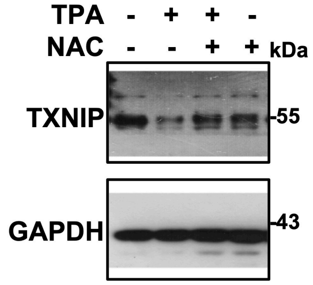

Figure 3I

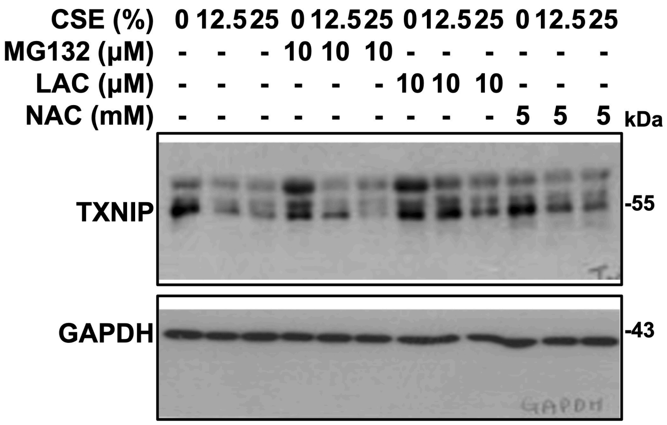

Supplementary Figure 4

Figure 4C

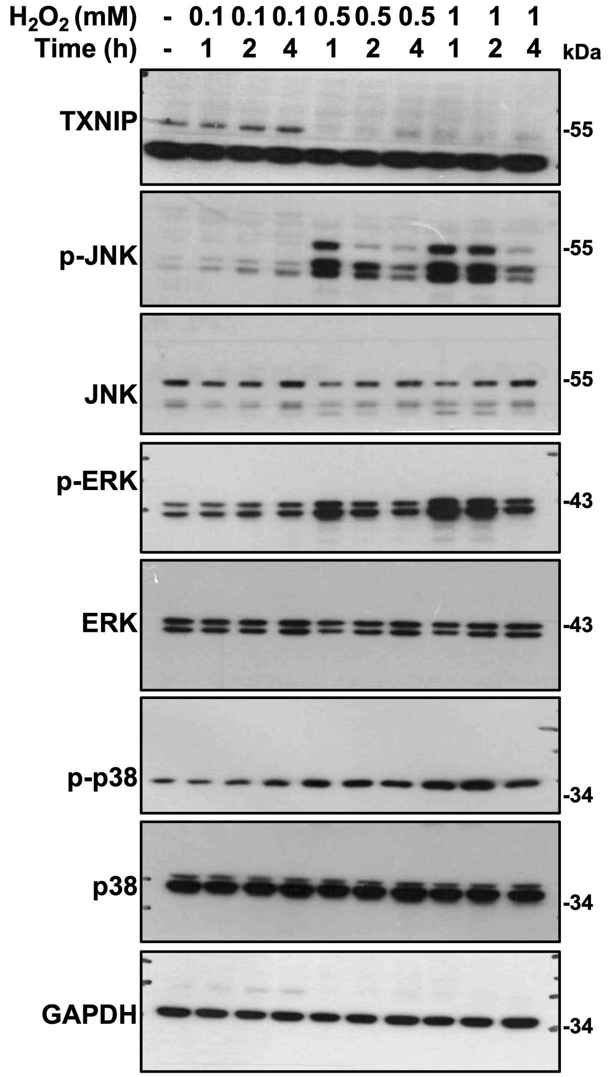

Figure 4D

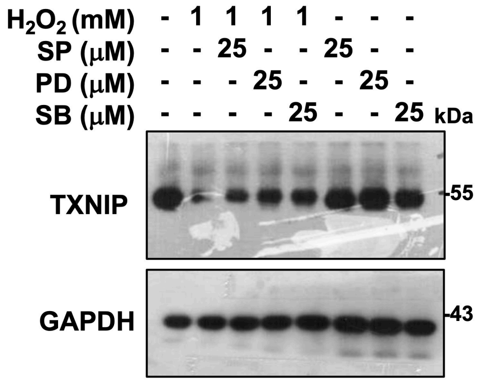

Supplementary Figure 5

Figure 5A

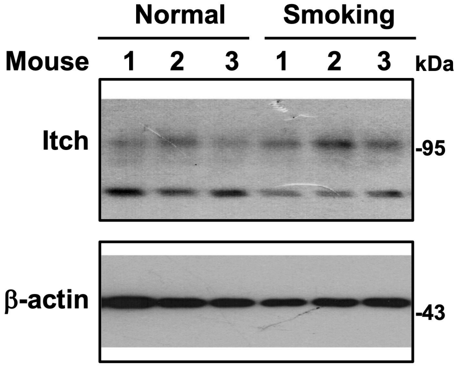

Figure 5B

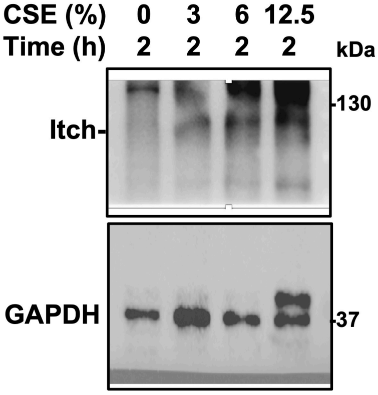

Figure 5C

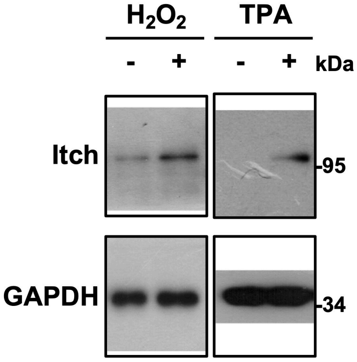

Figure 5D

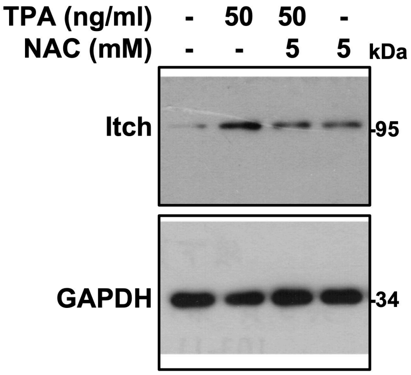

Figure 5E

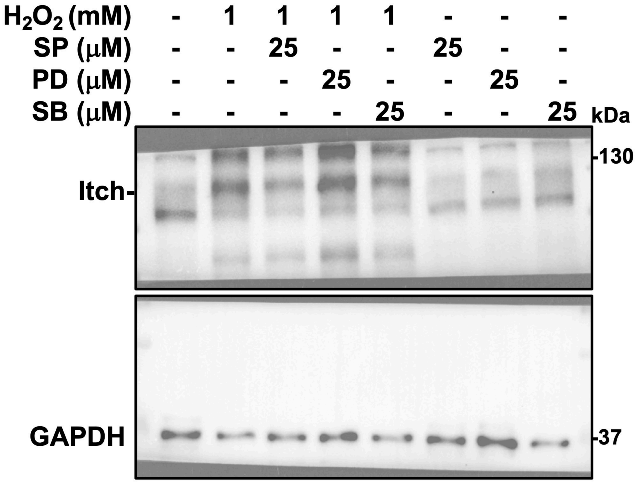

Figure 5F

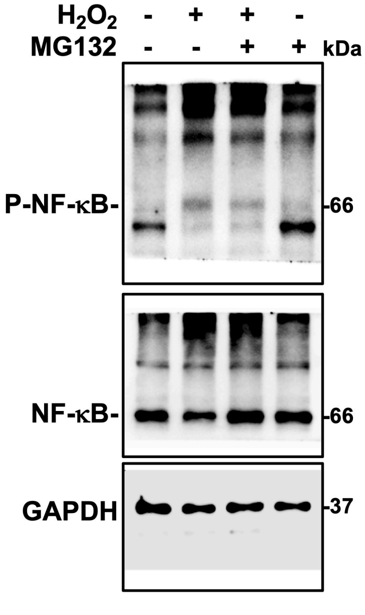

Figure 5G

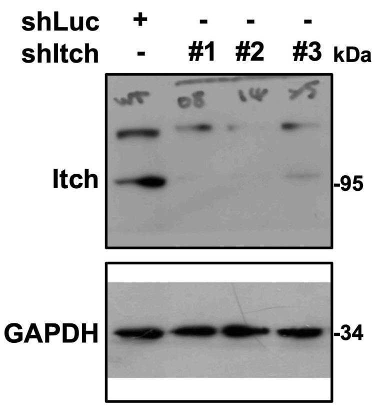

Figure 5H

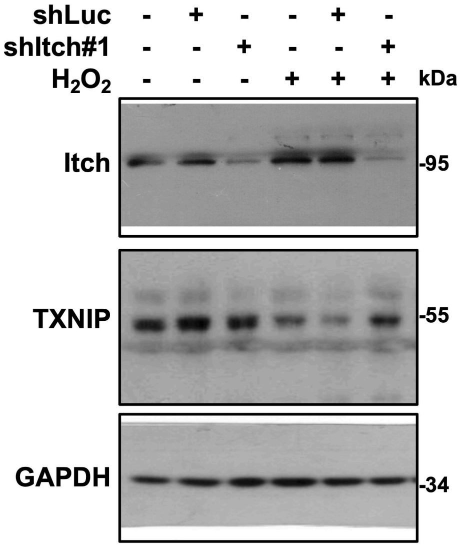

Supplementary Figure 6

Figure 6C

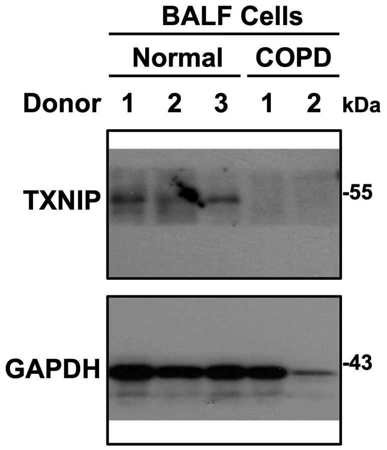

Figure 6D

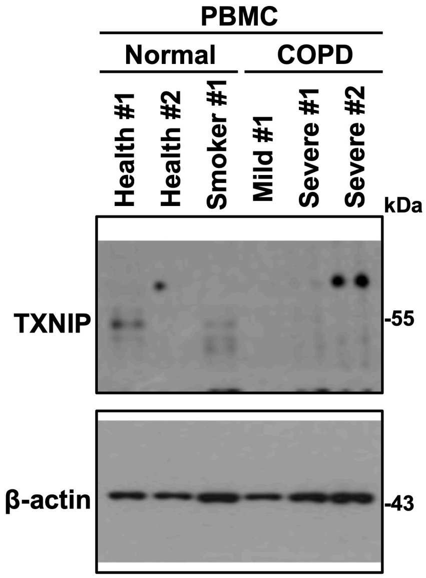

Supplement: Supplementary file 1 — Additional file 1. Raw western blot images. [file 12931_2025_3369_MOESM1_ESM.pdf]
